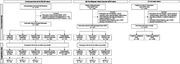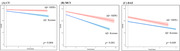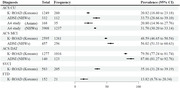# Clinical effects of amyloid positivity upon positron emission tomography in an Asian cohort with Alzheimer’s disease and other dementias

**DOI:** 10.1002/alz.089425

**Published:** 2025-01-09

**Authors:** Min Young Chun, Hyemin Jang, Jihwan Yun, Jun Pyo Kim, Sung Hoon Kang, Michael S. W. Weiner, Hee Jin Kim, Duk L Na, Chang‐Hyung Hong, Hyung Woong Roh, Tae‐Kyeong Lee, Eek‐Sung Lee, EUN HYE LEE, Daeun Shin, Hongki Ham, Yuna Gu, Yeshin Kim, Chi‐Hun Kim, Sook‐Young Woo, Sang Won Seo

**Affiliations:** ^1^ Samsung Medical Center, Sungkyunkwan University School of Medicine, Seoul Korea, Republic of (South); ^2^ Yonsei University College of Medicine, Seoul Korea, Republic of (South); ^3^ Yongin Severance Hospital, Yonsei University Health System, Yongin, Gyeonggi‐do Korea, Republic of (South); ^4^ Seoul National University Hospital, Seoul National University College of Medicine, Jongno‐gu, Seoul Korea, Republic of (South); ^5^ Samsung Medical Center, Sungkyunkwan University School of Medicine, Gangnam‐gu, Seoul Korea, Republic of (South); ^6^ Alzheimer's Disease Convergence Research Center, Samsung Medical Center, Seoul Korea, Republic of (South); ^7^ Korea University Guro Hospital, College of Medicine Korea University, Seoul Korea, Republic of (South); ^8^ University of California, San Francisco, San Francisco, CA USA; ^9^ Alzheimer’s Disease Convergence Research Center, Samsung Medical Center, Seoul Korea, Republic of (South); ^10^ Happymind Clinic, Seoul Korea, Republic of (South); ^11^ Ajou University School of Medicine, Ajou University Hospital, Suwon, Suwon Korea, Republic of (South); ^12^ Ajou Univeristy School of Medicine, San 5, Wonchon‐dong, Yeongtong‐gu, Suwon 443‐721 Korea, Republic of (South); ^13^ Soonchunhyang University Bucheon Hospital, Bucheon Korea, Republic of (South); ^14^ Soonchunhyang University Bucheon Hospital, Bucheon, Gyeonggi‐do Korea, Republic of (South); ^15^ Samsung Medical Center, Seoul Korea, Republic of (South); ^16^ Department of Neurology, Kangwon National University Hospital, Chuncheon Korea, Republic of (South); ^17^ Hallym University Sacred Heart Hospital, Anyang, Gyeonggi‐do Korea, Republic of (South)

## Abstract

**Background:**

Ethnic differences in amyloid‐β (Aβ) characteristics and cognitive trajectories should be considered, when designing Aβ‐targeted therapies. The clinical effects of co‐pathologies of Aβ have not been evaluated extensively across various dementias. We investigated the prevalence of Aβ+ and cognitive trajectories in Koreans and non‐Hispanic whites (NHWs) with Alzheimer’s clinical syndrome (ACS) and the clinical effects of Aβ+ in other dementias.

**Method:**

We included 5,856 Koreans who underwent Aβ positron emission tomography (PET) in South Korea. The participants were categorized into the following groups: ACS, including cognitively unimpaired (CU), mild cognitive impairment (MCI), and dementia of the Alzheimer type (DAT); subcortical vascular cognitive impairment (SVCI); and frontotemporal dementia (FTD). Data from 929 NHWs were collected from the Alzheimer’s Disease Neuroimaging Initiative. Mini‐Mental State Examination (MMSE) scores performed at least twice were used to compare cognitive trajectories. We performed multivariable analyses and stabilized inverse probability of treatment weights based on the propensity scores to mitigate imbalances across ethnic groups.

**Result:**

The prevalence of Aβ+ was lower in CU Koreans than in CU NHWs (odds ratio 0·6); however, this pattern was not observed in the MCI and DAT stages. Moreover, Aβ+ Koreans in the CU and MCI stages showed a more rapid decline in MMSE scores than Aβ+ NHWs (CU: B= ‐0·3136, p=0·004; MCI: B= ‐0·3847, p<0·001). Compared to CU, SVCI had a higher prevalence of Aβ+ in the 75–90 years age group (p<0·001). Aβ+ was predictive of a more rapid decrease in MMSE scores in SVCI (B= ‐0·6424, p<0·001) but not in FTD.

**Conclusion:**

Our findings contribute to understanding Aβ biomarker traits in various dementias in Asians and highlight distinct ethnic differences when compared to NHWs.